# The Surgical Lips Deformity Corrected with Hyaluronic Fillers: A Case Report

**DOI:** 10.3889/oamjms.2015.067

**Published:** 2015-07-02

**Authors:** Dragan Stolic, Maja Jankovic, Marija Draskovic, Slobodan Georgiev, Marina Stolic

**Affiliations:** 1 *Medica Aesthetica, Belgrade, Serbia*; 2 *Faculty of Medical Sciences, University of Kragujevac, Kragujevac, Serbia*; 3 *Dentaes, Skopje, Republic of Macedonia*

**Keywords:** Lip, hyaluron, implant

## Abstract

**BACKGROUND:**

Hyaluronic filler is a sterile, biodegradable, viscoelastic, isotonic, transparent injectable gel implant which was approved by Food and Drug Administration (FDA) 1996. It is used for face reconstruction and modelling.

**CASE PRESENTATION:**

We report the case of a 40-year-old Serbian woman who presented after surgery of cleft lip, primary and secondary palate. We performed a biphasic therapy; in the first stage in the zone semimucosis lips is initially carried incision scar tissue. The second stage is placed hyaluronan implant.

**CONCLUSION:**

This case illustrates that, although hyaluronic fillers used mainly for correction of healthy tissue can be successfully used in the treatment of postoperative scars.

## Introduction

In recent years there has been a growing interest in surgical procedures for facial rejuvenation. Hyaluronic acid is currently the most widely used dermal filler for the treatment of facial wrinkles [1]. Hyaluronic filler is a sterile, biodegradable, viscoelastic, isotonic, transparent injectable gel implant which was approved by Food and Drug Administration (FDA) 1996. It is used for face reconstruction and modeling. Aesthetic indications for lips are qualitative changes - shape, quantitative changes- size and biorevitalisation of lips, and reconstructive indications are asymmetry of lips, scars (postoperative, accidental, postinflamatory) and incompetent lips. The dermal fillers show an excellent tolerability and preservation of the dermal cells and matrix components [2].

Over the last decade, injectable soft tissue fillers have become an integral part of facial plastic surgery practice. The vast choice of new products being brought to the market, improved safety profile, lower costs in the current economic climate and high street availability mean that demand for nonsurgical rejuvenation treatments are increasing at an exponential rate and are no longer the preserve of the affluent [3].

We report a case of use of hyaluronic acid for the correction of postoperative scars after surgery cleft lip, primary and secondary palate.

## Case presentation

We report the case of a 40-year-old Serbian woman who presented after surgery of cleft lip, primary and secondary palate. She was born with palate shisis, lip shisis and nose deformity. She had more surgical interventions in order to take care of cleft palate.

**Figure 1:**
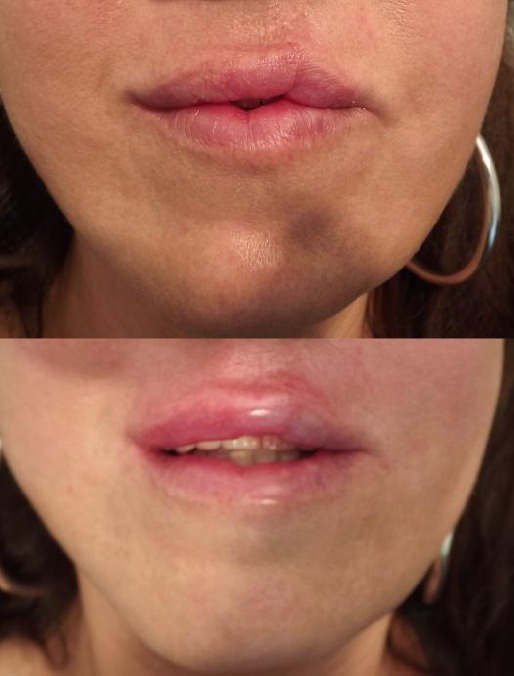
*Lips patients before and after treatment with hyaluronic fillers*.

Patient was admitted to the Aesthetic Education Center Medica Aesthetica in Belgrade, Serbia for aesthetic treatment after surgery. After clinical examination and specifying the desired look with a patient, correcting the application is accessed hyaluronic fillers. Preparing patients included the provision of short-term local infiltrative 2% lidocaine - epinephrine (lidocaine 40 mg/2 ml + epinephrine 0.025 mg/2 ml) plexus anesthesia, local anesthesia terminal branches of the mandibular nerve and disinfection of the operating field. We used a cross-linked hyaluronic acid (sodium hyaluronate) in the form of a gel concentration of 18.5 mg/g with the addition of antioxidants (mannitol) STYLAGE Special Lips, VIVACY laboratories, originating in France. Hyaluronic Filer is a non-animal origin, sterile and non-pyrogenic, physiological pH and osmolality. For injection is used needle 30G thickness, length 13 mm factory packed with hyaluronic fillers. When patients were done soft tissue reconstruction of the oral region, the application of hyaluronic acid injection was administered retrograde linear and bolus technique. We performed a biphasic therapy in the first stage in the zone semimucosis lips is initially carried incision scar tissue. We applied incision fibrous tissue release adhesions. The second stage is placed hyaluronan implant linear retrograde technique and bolus technique whereby the injected 0.33 ml of material. In order to achieve the most natural-looking lips in accordance with the accepted average proportions lips were drawn contours and minimum compensation amount of lip volume of 0.5 ml hyaluronic fillers (Figure 1).

## Discussion

Soft tissue augmentation with temporary dermal fillers is a continuously growing field, supported by the ongoing development and advances in technology and biocompatibility of the products marketed [1, 2]. The longer lasting, less immunogenic and thus more convenient hyaluronic acid (HA) fillers are encompassing by far the biggest share of the temporary dermal filler market [4-6].

The after multiple surgeries innate cleft palate, usually followed by corrective intervention of plastic surgeons. This requires hospitalization of patients, postoperative swelling and a longer recovery. Our patients wanted painless, comfortable treatment to correct the aesthetic defect. The case highlights the importance of proper injection technique, as well as the need for immediate recognition and treatment of similar scars [7]. The combination of treatments with fillers and surgical procedures may help process and provide more natural results than are possible with any of these techniques alone [8].

The HA dermal filler was associated with minimal discomfort, bruising or swelling of the lips; almost two-thirds of subjects (62%) returned to social engagements on the same day [4]. The high degree of subject satisfaction with aesthetic improvement in the lips, as well as the natural look and feel, indicates that this HA dermal filler represents an effective treatment option for patients requiring lip enhancement [9].

Injection of synthetic fillers for soft tissue augmentation is increasing over the last decade. One of the most common materials used is hyaluronic acid (HA) that is safe and temporary filler for soft tissue augmentation [7, 10]. It is imperative to any technique that direct, and preferably quantitative, feedback is given so that an immediate modification can be generated and successive patient outcomes improved [11]. Soft-tissue augmentation of the face is increasingly popular and the number of available filling agents has increased dramatically, improving the range of options for HA fillers. Meta-analysis proved both safety and efficacy for HA fillers [12].

Replacement fillers (such as the various formulations of HA) provide space-filling volume for a finite period of time [13]. Our expanding understanding of the physiological and immunological conditions of the skin and has prompted a growing field of aesthetic technology. Restorative procedures are taking advantage of improved and refined biotechnology, which continues to evolve at a rapid pace. Whereas surgical correction of skin laxity was the norm in years past, there are now many topical options available, and an ever-growing, increasingly perfected depot of minimally invasive, injectable dermal volumizers and stimulators, collectively referred to as dermal fillers [14]. Use of dermal and subdermal fillers for facial rejuvenation has become popular because these treatments provide desirable aesthetic outcomes such as a harmonious, attractive appearance without invasive surgical procedures and without the downtime associated with surgery. While no procedure is free from risk to the patient, the appropriate use of fillers is generally associated with lower risk and less downtime compared with surgery [15].

In conclusion, this case illustrates that, although hyaluronic fillers used mainly for correction of healthy tissue can be successfully used in the treatment of postoperative scars.
